# Ecological and social factors lead to variation in parental care between sexes in a burying beetle

**DOI:** 10.1111/1744-7917.70094

**Published:** 2025-06-24

**Authors:** Donghui Ma, Long Ma, Jan Komdeur

**Affiliations:** ^1^ Groningen Institute for Evolutionary Life Sciences (GELIFES) University of Groningen Groningen the Netherlands; ^2^ GIGA‐Neurosciences, GIGA Institute University of Liège Liège Belgium

**Keywords:** burying beetle, interspecific competition, intraspecific competition, parental care, resource availability, sexual conflict

## Abstract

Sexual conflict over parental care represents a divergence in the evolutionary interests between males and females, consequently leading to distinct sex roles in parental care and reproductive strategies. However, whether and how various ecological and social environments influence such sex differences remains largely unclear. In this study, using the burying beetle *Nicrophorus vespilloides*, which breed on carcasses and exhibit biparental care pattern, we investigated the impacts of resource availability, intraspecific and interspecific competition on individual parental care and reproduction in males and females. We manipulated the resource availability for each breeding pair by providing either a large or a small mouse carcass. Furthermore, we constructed the presence of intraspecific and interspecific competition for pairs breeding on large carcasses, by introducing an additional, small pair of beetles and blowfly maggots *Calliphora*, respectively. We found that, compared with males and females breeding on large carcasses, males did not change their care, whereas females increased their care when breeding on small carcasses. In the presence of another pair of beetles, both males and females of the focal pair increased their care, whereas in the presence of blowfly maggots, they decreased their care. Our results showed that males and females adjust their parental care based on resource availability and competition pressure, with sex‐specific differences driven by ecological and social factors. Our study sheds light on the importance of sex‐specific parental care in response to various external drivers, which contributes to a better understanding of the evolution of sexual conflict over parental care.

## Graphical abstract

We experimentally investigated the effects of ecological (resource availability and interspecific competition) and social factors (intraspecific competition) on parental care and reproductive success in the burying beetle *Nicrophorus vespilloides*. Our results showed that males and females adjust their parental care based on resource availability and competition pressure, with sex‐specific differences driven by ecological and social factors.

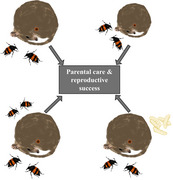

## Introduction

Sexual conflict is a fundamental and long‐standing topic in behavioral ecology, which refers to an antagonistic selection process acting on two sexes with different interests (reviewed by Arnqvist & Rowe, 2005). This conflict arises when males and females face unmatched or opposing selection on costly reproductive traits and behaviors (e.g. parental care behavior), causing divergent optimal fitness benefits for each sex (Parker, 1979; Arnqvist & Rowe, 2005; Bonduriansky *et al.*, 2008; Lessells, 2012; Taborsky *et al.*, 2014; Paquet & Smiseth, 2016). “Anisogamy” theory has been proposed to explain the different sex roles and their consequent effects on mating and parental care, suggesting that the asymmetry between sexes may emerge from gametes, which may extend to parental care at the pre‐ and postnatal stages of offspring (Trivers, 1972). Sexual conflict over parental care can be considered as a tug‐of‐war interaction between sexes, where both parents share benefits from their combined parental care to their young, whereas the costs of care are paid by each parent due to its own care to raise the young. Consequently, it could be expected that each parent is under selection to minimize its individual care by grafting more parental workload over to its partner (Arnqvist & Rowe, 2005; Houston *et al.*, 2005; Székely *et al.*, 2014; Paquet & Smiseth, 2016). Understanding how sexual conflict is resolved is critical, as it eventually shapes the emergence and stability of biparental care as an Evolutionary Stable Strategy (Houston *et al.*, 2005; Harrison *et al.*, 2009; reviewed by Paquet & Smiseth, 2016).

Theoretical studies have proposed three potential types of behavioral interactions between male and female parents that may explain the resolution of sexual conflict over parental care (Johnstone & Hinde, 2006; Lessells, 2012; Paquet & Smiseth, 2016), including negotiation, matching, and sealed‐bid responses. Negotiation models assume that each parent directly adjusts its level of care in response to its partner's care, and that the focal parent may partially compensate for decreased care by its partner (McNamara *et al.*, 1999). However, matching models highlight that each parent adjusts its level of care to match any increase or reduction in the partner's contribution (Johnstone & Hinde, 2006). Sealed‐bid models assume that the parental contribution of each individual is fixed and not influenced by its partner (Houston & Davies, 1985). Empirical works across animal species (almost all in avian species) provide evidence for all three models, where the two parents do not always deploy similar behavioral strategies to respond to the workload of their partners (Schwagmeyer *et al.*, 2002; Johnstone & Hinde, 2006; Harrison *et al.*, 2009). For example, females of Great Tits *Parus major* fully compensate for their partner's decrease in feeding rates, whereas males do not show any compensation and even tend to decrease their feeding rates (Sanz *et al.*, 2000). However, the extent to which such behavioral strategies are influenced by ecological and social factors remains less explored (Wong & Candolin, 2015; Des Roches *et al.*, 2018).

Across and within species, evolutionary variation in magnitude and forms of parental care is driven by sex‐specific costs and benefits, which is also the result of changes in ecological and social environments experienced by males, females, or both sexes (e.g. van Dijk *et al.*, 2012; Zheng *et al.*, 2018; Ma *et al.*, 2021; Long *et al.*, 2022). Normally, individuals adjust their decisions in parental care according to the variation of internal factors (e.g. body condition, age, and the level of hormones; Achorn & Rosenthal, 2020) and external factors (e.g. ecological and social environments; Ma *et al.*, 2021). For example, parents with good body conditions are likely to invest more time and energy in offspring care, maximizing reproductive advantages, compared with those with poor body conditions (Achorn & Rosenthal, 2020). This is because poor body conditions may lead to constrained resources available for self‐maintenance and future breeding opportunities (Soulsbury, 2019; Pontzer & McGrosky, 2022). As an example of external factors, resource availability, such as food abundance and territory quality, can affect individual parental care and reproduction. Individuals that breed in a high‐quality territory may forage more efficiently, gain more food resources for breeding, and may increase their parental care, compared with those who breed in a lower‐quality territory (Gauthier & de Jong, 2021). As another external factor, the pressure of competition with intraspecific and interspecific individuals is also found to directly influence reproductive success, which also alters an individual's parental care and reproduction (e.g. Requena *et al.*, 2009; Grayson *et al.*, 2013; Chan *et al.*, 2019; Ratz *et al.*, 2022).

In this study, we investigated the effects of ecological and social factors on male and female parental care and examined whether and how parents coordinate their care to mitigate sexual conflict across these varying contexts, using the burying beetle *Nicrophorus vespilloides* as a model species. Burying beetles typically exhibit intricate parental care, which provides an ideal biological system for studying the mechanisms of the co‐evolution of parental care between sexes across animal species, especially in insects, and their roles in response to the changes in environmental and social stressors (Paquet & Smiseth, 2016; Potticary *et al.*, 2024). Burying beetles use small vertebrate carcasses as breeding and food resources that support food for offspring and parents themselves (Scott, 1998). These beetles often breed as pairs, jointly preparing the carcasses and preserving them with antimicrobial secretions (Scott, 1998; Smiseth *et al.*, 2005; Steiger *et al.*, 2007). Females lay eggs in the soil near the prepared carcass, and together with the males, provide post‐hatching care for the developing larvae, such as food provisioning. Parents cooperatively defend the broods against intruders and feed the developing larvae on the prepared carcass. However, in some cases, both males and females can rear larvae alone (e.g. Trumbo, 1994; Smiseth & Moore, 2004; Paquet *et al.*, 2017; Ratz *et al.*, 2020). Carcasses are ephemeral, unpredictable resources for beetles, their extent of decomposition and size highly determine the reproductive success of beetles and influence their parental care into current reproduction (e.g. Richardson & Smiseth, 2019; Wang *et al.*, 2022). Furthermore, parental care and investment is also influenced by other factors, such as individual body size and nutritional condition (e.g. Pilakouta *et al.*, 2015; Richardson & Smiseth, 2019), loss of mate (Smiseth *et al.*, 2005), brood size (Wang *et al.*, 2021), competitive interactions with intruders (Ma *et al.*, 2022), and interactions with parasites (e.g. mites) (De Gasperin *et al.*, 2015). Although it has been suggested that females primarily provide direct care by provisioning larvae, both sexes are found to contribute to carcass maintenance and brood defense against intruders (Scott, 1990; Eggert *et al.*, 1998; Cotter & Kilner, 2010; Wang *et al.*, 2021; Wang *et al.*, 2022). Female burying beetles always exhibit higher levels in many aspects of parental care compared with males, whereas both females and males show a sex difference in adjusting parental care in response to mate loss (Scott, 1990; Müller *et al.*, 1998; Smiseth *et al.*, 2005; Cotter & Kilner, 2010). For example, in *N*.* vespilloides*, both males and females contribute to social immunity of broods by secreting antibacterial oral and anal exudates, while they show a sex‐specific adjustment in such antibacterial activity in response to mate loss (Cotter & Kilner, 2010).

In this study, we manipulated resource availability during breeding by providing beetle pairs with either large (ca. 25 g, i.e. control group) or small (ca. 15 g, i.e. small carcass group) carcasses. To investigate the impacts of intraspecific competition (i.e. intraspecific competition group), we constructed the presence of intraspecific competition by introducing a second pair of beetles to a pair of beetles breeding on a single, large carcass. To create the pressure of interspecific competition, pairs of beetles were provided with a flyblown, large carcass, simulating natural competition with blowflies, one of the major reproductive rivals that negatively affects the discovery and quality of carcasses and subsequent reproductive success (Bartlett, 1987a; Scott, 1998; Sun *et al.*, 2014; Chan *et al.*, 2019). We quantified parental care by recording the frequency of parental presence on or within the carcass, without differentiating specific behavior. While such behavioral measures combine direct care (e.g. larval provisioning), indirect care (e.g. carcass maintenance and protection), and self‐maintenance behavior, this behavioral measure can reflect overall parental investment during the whole breeding period (Wang *et al.*, 2021; Ma *et al.*, 2022; Wang *et al.*, 2022). Specifically, parental care of the male and female of the pair, and the total parental care of the pair (male and female combined) was measured. We also collected data on reproductive success, including brood size, brood mass, and the number of newly eclosed beetles, as well as changes in parental body mass, to assess the effects of parental care under varying ecological and social conditions.

There are 4 main predictions. First, we predicted males under resource limitation (i.e. small carcasses) would decrease their parental care, whereas females might maintain or increase their parental care. This difference may be due to increased competition for resources between the two parents, as well as between parents and offspring (Bartlett, 1987a; Müller *et al.*, 1990b; Richardson & Smiseth, 2019), despite a potentially reduced parental investment in resource maintenance and defense for both parents (Wang *et al.*, 2022). For example, *Nicrophorus orbicollis* males have been shown to exhibit shorter parental care duration on smaller carcasses (Scott & Traniello, 1990). However, females may benefit from male desertion (Boncoraglio & Kilner, 2012), and male contributions do not always enhance offspring fitness under laboratory conditions beyond correlational findings (Parker *et al.*, 2015; but see Pilakouta *et al.*, 2018). These studies demonstrate an increased sexual conflict over resources and parental care between parents under limited resource conditions, which may also be a factor that underpins the evolution of a female uniparental care system in burying beetles (Boncoraglio & Kilner, 2012; but see Benowitz & Moore, 2016). Second, in the presence of intraspecific competition, we expected that both males and females would increase their parental care, although such changes may be inconsistent. In the presence of intraspecific individuals (e.g. co‐breeding scenario), both males and females may benefit from enhanced parental investment in brood care and defense and increased access to the carcass, such as low risks of infanticides by co‐breeders, and increased resource saving for future reproduction (Bartlett, 1987b; Müller & Eggert, 1990; Komdeur *et al.*, 2013; Ma *et al.*, 2022). However, in such scenarios, reproductive benefits and costs of parental care are different for males and females, for example, extra‐pair copulations for males (Richardson & Smiseth, 2020; Ma *et al.*, 2022). Third, we expected that in the presence of interspecific competition by blowflies, both males and females would similarly increase their parental care. Blowflies are a common rival for both males and females, as their presence causes detrimental impacts on carcass utilization and reproductive benefits (Bartlett, 1987a; Scott, 1998; Sun *et al.*, 2014; Chan *et al.*, 2019). Finally, following these predictions, we anticipated that adjustment of parental care and parental coordination over care between sexes (i.e. the correlation between male and female care) would vary across ecological and social contexts. Our research will contribute toward a better understanding of the evolution of parental care and help to understand the eco‐evolutionary factors that facilitate sexual conflict.

## Materials and methods

### Burying beetle husbandry

Adult beetles used for this study descended from F_2_ to F_5_ generations of an outbred laboratory population at the University of Groningen. The laboratory population was derived from wild‐caught beetles at the estate De Vosbergen, Eelde, the Netherlands (53°08ʹ N, 06°35ʹ E), from May to August in 2017 and 2018, using pitfall traps baited with chicken livers. During the entire rearing period, up to 6 same‐sex adult beetles derived from the same brood were kept in rearing boxes (10 cm length × 6 cm width × 8 cm height) with 2‐cm depth of wet peat. They were fed twice a week with 1−2 mealworms per beetle. Throughout the experiment, all adults and offspring were maintained at a temperature of 20 °C with a 16 h light : 8 h dark photoperiod (Wang *et al.*, 2021; Ma *et al.*, 2022; Wang *et al.*, 2022). Experimental conditions replicated the mean temperature and photoperiod of Groningen, the Netherlands, during the natural breeding season (May−August).

### Experiment protocol

In this study, we designed three independent experimental groups and a control group, to explore the effects of resource availability, and intraspecific and interspecific competition on the degree of sexual conflict over parental care in burying beetles. Sexually mature, virgin adult beetles, aged approximately 2 weeks at post‐eclosion, were selected for this experiment. Just before the experiment, the body size of each individual was recorded by measuring the pronotum width using an electric caliper (accuracy: 0.01 mm) (Beeler *et al.*, 2002; Hopwood *et al.*, 2016), and weight was measured using an electric scale (accuracy: 0.0001 g). Males and females were individually marked by pricking, without injuring the beetles, 2 small holes in one of the elytra (i.e. the left side for males and the right side for females) using an insect pin. No beetles died accidentally after the marking. This method allowed us to recognize the parental individuals during the subsequent daily inspections of the breeding boxes. For each breeding event, each beetle was paired with a similarly sized (i.e. the difference in body size is limited to 0.1 ± 0.03 mm), unrelated partner, and housed with its mate into a rearing box with clean peat for 12 h, to make sure that females had been fertilized (Wang *et al.*, 2021; Ma *et al.*, 2022; Wang *et al.*, 2022). Then, each pair was transferred into a breeding box (23 cm length × 19 cm width × 12.5 cm height) with 3−5 cm of moist peat, and given a thawed, previously frozen, dead mouse as breeding resource.

To create control (*n* = 52) and small carcass (*n* = 47) treatments, beetle pairs were given a large carcass (ca. 25 g) or a small carcass (ca. 15 g) respectively. These sizes were selected because these carcasses could be used by this species under both natural and laboratory conditions (Scott, 1998; Smiseth & Moore, 2008). To create the intraspecific competition treatment (*n* = 78), we placed an additional pair of beetles as intruders at 12 h after the resident pair was provided with a ca. 25‐g carcass for breeding. These additional pairs were smaller in body size (ca. 10%−15% size difference) compared with the resident breeding pairs. This could potentially decrease the harmful risks from intruders to the residents and the brood, such as the usurpation of carcasses by intruders, because an individual's body size largely determines its competitive ability and intense fights were less likely to occur between opponents with a larger size difference in burying beetles (Otronen, 1988; Beeler *et al.*, 2002; Komdeur *et al.*, 2013). To construct the interspecific competition treatment (*n* = 33), we created the “blowfly maggots usurped” carcass (ca. 25 g), by making a small incision (1‐cm diameter) to open the abdomen of a thawed mouse carcass using a scalpel, and then introducing 2.0 ± 0.2 g newly hatched blowfly maggots (*Calliphora*) into the carcass, then give it to a beetle pair for breeding. To maintain a continuous pressure of interspecific competition, the introduction of 2.0 ± 0.2 g newly hatched blowfly maggots into carcasses was repeated 24 h after each beetle pair received the “blowfly maggots usurped” carcass. Based on our treatments, the control group and the other three experimental groups differed in only 1 factor, which is either resource availability, or intraspecific and interspecific competition pressure.

### Behavior observation and data collection

Across all treatment groups, parental care, body mass change of each beetle, and reproductive success were measured. During the entire period of each breeding event (from the onset of the experiment until larval dispersal from the carcass), we checked each breeding box three times daily (7:00−9:00 a.m., 2:00−4:00 p.m., 9:00−11:00 p.m., 5‐h intervals) by visual inspection (instant scanning) through carefully removing the surface soil of the carcass. During each check, we observed beetles for 15 s per group continuously, and recorded for each beetle whether or not it was on the carcass, as an indicator of parental care in burying beetles (Wang *et al.*, 2021; Ma *et al.*, 2022; Wang *et al.*, 2022). After each check, we carefully re‐covered the carcass with soil. We defined parental care behavior as an individual providing indirect parental care (i.e. carcass guarding and maintenance on the surface of the carcass), and/or direct parental care (i.e. larvae provisioning inside the carcass) (Smiseth *et al.*, 2005). We calculated individual parental care as the frequency of the focal parent being present on or within the carcass of the total checked times (i.e. individual parental care = times of parent being present/times of inspecting breeding boxes). Once the larvae dispersed, all alive parents were weighed to calculate the body mass change of adults over the breeding events (i.e. body mass change = [final mass – initial mass]/initial mass), which is seen as an indicator of parental investment toward current reproduction (Wang *et al.*, 2021; Wang *et al.*, 2022). We also recorded the number of dispersed larvae (hereafter brood size) and weighed them in broods (hereafter brood mass), and the number of newly eclosed beetles after pupation (hereafter offspring number) for evaluating the reproductive success of each brood.

### Data analyses

All statistical tests were performed using R version 4.2.1 (R core team, 2022) loaded with packages “car” (Fox & Weisberg, 2019), “lme4” (Bates *et al.*, 2015), “MASS” (Venables & Ripley, 2002), and “emmeans” (Lenth, 2023). We used a *cbind* function to combine times of male (i.e. *T*_male_) or female (i.e. *T*_female_) being present on or in the carcass, with the times that we checked the breeding boxes (i.e. *T*_check_) subtracting times of the parent being present on or in the carcass as response variables, that is, *cbind* (*T*_(fe)male_, [*T*_check_ – *T*_(fe)male_]). Firstly, we performed binomial generalized linear mixed models (GLMMs) to compare the care of males and females under our treatments, in which we set the treatment, parental sexes, and their interaction as fixed factors, and the breeding box ID as a random factor. Second, we performed similar GLMs for the total parental care of a pair, where the response variable follows the formula: *cbind* ([*T*_male_ + *T*_female_], [2 × *T*_check_ – *T*_male_ – *T*_female_]), and the explanatory variable is the treatment. Third, to examine the effect of social and ecological factors on parental coordination over parental care, we performed binomial GLMs for the ratio between male and female parental care (i.e. male care/female care). The response variable was expressed using the formula *cbind* (*T*_male_, *T*_female_), with the explanatory variables being the treatment and *T*_check_. Additionally, we tested the correlation coefficient between male and female care under the different treatments using *cor*.*test* function with a Pearson's method. Finally, we used negative binomial GLMs to test brood size and offspring number (integer values), and linear (mixed) models to test brood mass (LMs) and parental body mass change (LMMs). We set the treatment, male and female care (i.e. [*T*_male_/*T*_check_] and [*T*_female_/*T*_check_]) as explanatory variables. Furthermore, in the models for parental body mass change, we also included the sex of parents, and its interaction with the treatment as explanatory variables, and the breeding box ID as a random factor. We used the *emmeans* function to perform *post hoc* Tukey's correction tests when the interaction effects and the treatments showed a significant effect on targeted response variables. In these cases, we compared the three treatment groups with the control using the *trt*.*vs*.*ctrl* method. We applied the *summary* function to show and report the results of models. Figures were generated using the “*ggplot2*” package (Wickham, 2016).

## Results

### Parental coordination over care between sexes based on resource availability, intraspecific and interspecific competition

**Fig. 1:**
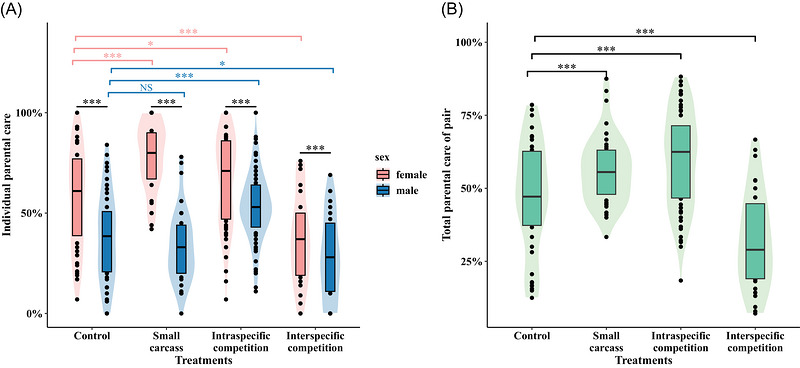
Comparison of (A) parental care of male and female individuals and (B) total parental care of pairs under experimental treatments. Black dots indicate outliers. The significance of the differences was determined using a *post hoc* test following the generalized linear (mixed) models analysis. In (A), black annotation lines denote comparisons of parental care between males and females within the same treatment groups; blue lines indicate comparisons of male parental care in the control group with the other three treatment groups, whereas pink lines represent comparisons of female parental care in the control group with the other three treatment groups. Significance code: NS, *P *> 0.05, *P < 0.05, **P < 0.01, ***P < 0.001.

The result of the GLMM analysis revealed that individual parental care was significantly influenced by the interaction between the treatment (i.e. control [large carcass], small carcass, intraspecific competition, and interspecific competition) and parental sexes (*χ* ^2^ = 74.27, *P* < 0.001; Table 1). Specifically, across all treatments, females provided more parental care than males. Compared with males and females in the control group, males did not change their parental care in the small carcass group, whereas females increased their parental care. Under intraspecific competition, both males and females increased their parental care compared with those in the control group, whereas under interspecific competition they decreased their parental care compared with those in the control group (Fig. 1A).

**Table 1:** Comparison of the control group with the other three groups was conducted for individual parental care of males and females, total parental care of pairs, and ratio of male and female care from the *post hoc* test of generalized linear (mixed) models

Traits	Estimate	Standard error	*Z* value	*P* value
Parental care of individuals				
Control: male–female	0.83	0.11	7.53	< 0.001
Small carcass: male–female	2.03	0.16	12.89	< 0.001
Intraspecific competition: male–female	0.56	0.09	6.42	< 0.001
Interspecific competition: male–female	0.51	0.13	3.81	< 0.001
Male: control–small carcass	0.16	0.18	0.88	0.690
Male: control–intraspecific competition	0.68	0.15	4.51	< 0.001
Male: control–interspecific competition	− 0.54	0.19	− 2.85	0.012
Female: control–small carcass	1.04	0.19	5.45	< 0.001
Female: control–intraspecific competition	0.40	0.15	2.67	0.022
Female: control–interspecific competition	− 0.87	0.19	− 4.70	< 0.001
Total parental care of pairs				
Control–small carcass	0.32	0.09	3.76	< 0.001
Control–intraspecific competition	0.46	0.07	6.96	< 0.001
Control–interspecific competition	− 0.65	0.08	− 8.02	< 0.001
Ratio of male and female care				
Control–small carcass	− 0.43	0.12	− 3.50	0.001
Control–intraspecific competition	0.19	0.09	2.03	0.121
Control–interspecific competition	0.08	0.13	0.65	0.889

From the perspective of total parental care provided by pairs, the result of the GLM analysis showed significant differences among treatments (LR*χ* ^2^ = 245.45, *P* < 0.001; Table 1). Specifically, compared with total parental care in the control group (large carcass), total parental care was significantly higher in the small carcass and the intraspecific competition groups, whereas total parental care was lower in the interspecific competition group (Table 1, Fig. 1B).

**Fig. 2:**
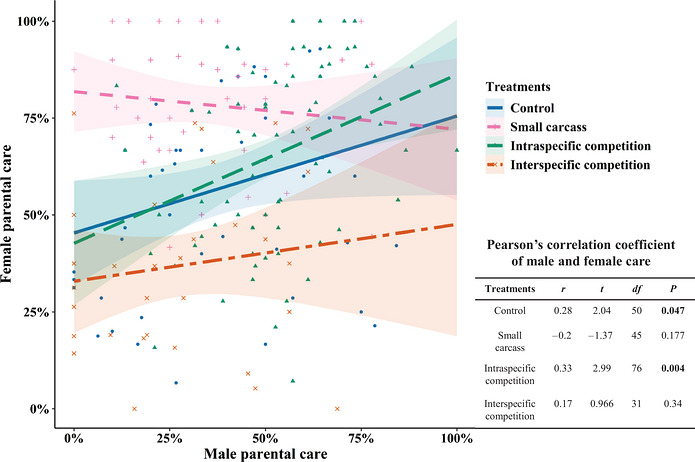
The correlation between male and female parental care in burying beetles under the 4 treatments. The colored regression lines represent the relationship for each treatment (± 95% confidence intervals).

The result of the GLM analysis for the ratio of male and female parental care (male care/female care) showed a significant effect of the treatment (LR*χ* ^2^ = 33.05, *P* < 0.001; Table 1). In detail, compared with the control group (large carcass; mean ± standard error [SE] = 0.85 ± 0.12), a significantly lower ratio of male and female parental care was found in the small carcass group (mean ± SE = 0.47 ± 0.04), indicating a greater difference in parental care between sexes with lower resource availability. In contrast, comparable ratios of male versus female parental care were observed in the intraspecific competition group (mean ± SE = 0.97 ± 0.10) and in the interspecific competition group (mean ± SE = 1.07 ± 0.30; Table 1, Fig. 1B). Furthermore, in the control and intraspecific competition groups, we found a significantly positive correlation in individual parental care between males and females, whereas in the small carcass and interspecific competition groups, male and female parental care were not correlated (Fig. 2).

### The influence of resource availability, intraspecific and interspecific competition on reproductive success and parental body mass change

**Fig. 3:**
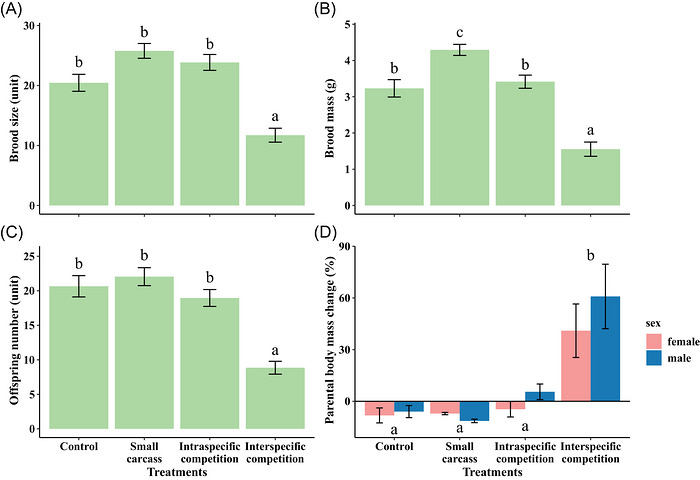
The difference in (A) brood size, (B) brood mass, (C) offspring number, and (D) parental body mass change between the experimental treatments. Identical letters obtained from *post hoc* tests indicate no significant difference, while different letters indicate a significant difference.

The results of GLM and LM analyses revealed that the treatment had significant effects on brood size, brood mass, and offspring number (Table 2). Compared with the control group (large carcass), similar brood size, similar offspring number, but heavier dispersed larvae, were found in the small carcass group (Fig. 3A–C). For the intraspecific competition group, all three components of reproductive success were similar to those for the control group (Fig. 3A–C). Conversely, breeding pairs in the interspecific competition group produced smaller brood size, lighter brood mass, and fewer newly eclosed beetles than breeding pairs in the control group (Fig. 3A–C). We found that neither male nor female care influenced brood size and brood mass. However, female care, but not male care, had a significantly positive effect on the number of newly eclosed offspring (Table 2).

**Table 2:** Effects of the experimental treatment and male and female parental care, on brood size, brood mass, and offspring number

Explanatory variables	Brood size			Brood mass			Offspring number		
	LR*χ* ^2^	df	*P* value	F	df	*P* value	LR*χ* ^2^	df	*P* value
Treatment	15.47	3	0.001	8.92	3	< 0.001	13.08	3	0.004
Male care	1.02	1	0.312	2.71	1	0.102	0.10	1	0.753
Female care	2.78	1	0.095	2.10	1	0.15	4.30	1	0.038

We found from the LMM analysis that the treatment (*F* = 18.87, *P* < 0.001) and parental sex (*F* = 10.81, *P* = 0.001) had significant effects on the parental body mass change, but their interaction did not (*F* = 0.512, *P* = 0.674). Compared with males and females of pairs in control group, males and females of pairs in the interspecific competition group showed more weight gain after breeding, whereas weight change of males and females was similar for pairs in the small carcass and the intraspecific competition groups (Fig. 3D). Furthermore, males have gained more weight after breeding than females (Fig. 3D). Additionally, for both males and females, weight gain during the breeding period was positively associated with the amount of parental care (*F* = 6.09, *P* = 0.002).

## Discussion

Our study reveals significant effects of variation in ecological and social environments on parental care and reproductive success among the burying beetle *N*.* vespilloides*. Specifically, beetle pairs breeding on small carcasses exhibited higher levels of total parental care provided by pairs compared with those breeding on large carcasses. This difference may be attributed to increased female care, because females, but not males, showed more parental care on small carcasses than on large carcasses. In the presence of intraspecific competition, both males and females elevated their parental care, resulting in higher levels of total parental care. Conversely, interspecific competition led to a reduction in care from both sexes, with lower levels of total parental care compared with those who breed without competitors (i.e. the control group). These findings highlight sex‐specific differences in parental care adjustments in response to ecological and social factors. Coordination in parental care was context‐dependent. Both sexes responded to the care of their partner by adjusting their care, with a positively correlated effort, when breeding on large carcasses and in the presence of intraspecific competition. In contrast, neither males nor females had a response to the change of their partner's care, when breeding on small carcasses and in the presence of interspecific competition. Furthermore, we found the significant effects of ecological and social factors on reproductive success and parental body mass change during breeding. Beetle pairs breeding in the interspecific competition group exhibited the lowest reproductive success, despite all parents gaining more body mass after breeding. In contrast, the other three groups (i.e. the control, small carcass, and intraspecific competition groups) had no significant difference in reproductive success and parental body mass change. These findings suggest that males and females respond differently to ecological and social environments, potentially reflecting sex differences in the costs and benefits of reproduction across contexts.

### Males and females adjusted parental care in relation to resource availability

Consistent with our prediction, females breeding on small carcasses increased their care for developing larvae compared with females in the control group, which was associated with heavier brood mass. However, contrary to our prediction, males maintained similar levels of care regardless of carcass size. One possible explanation is that females, but not males, are under stronger selective pressure to enhance their parental investment to secure reproductive success when resources are limited (Clutton‐Brock, 1991; but see Ma *et al.*, 2021). Carcass size preference in burying beetles depends on multiple factors, and the sizes we used may not represent adequate versus constrained conditions for breeding (Hopwood *et al.*, 2016; Hsu *et al.*, 2024). Although large carcasses are generally advantageous, their exploitation and preparation may be less efficient depending on the beetles’ body size. Our findings contrast with previous studies showing that male care tends to increase with carcass size, whereas female care remains unchanged (Ratz *et al.*, 2021; Wang *et al.*, 2022). This also demonstrates that in our study, the selection of beetles from size‐specific groups, rather than random assignment, may have influenced the patterns of care for females and males, as previous work has shown body‐size‐dependent parental care in females but not in males (Pilakouta *et al.*, 2015; Hsu *et al.*, 2024). Future studies should manipulate both carcass size and beetle size to clarify their interactive effects on parental care.

For burying beetles, the carcass size and its decomposition status represent the current environmental pressures that influence their reproduction and their decisions on parental investment, which also impacts the parental body maintenance and larval growth directly (e.g. Smiseth *et al.*, 2014; Richardson & Smiseth, 2019; Wang *et al.*, 2021; Wang *et al.*, 2022). Large carcasses could offer more resources for parents and offspring, potentially leading to a low level of sexual conflict between sexes due to their unrestricted access to the carcasses (Scott, 1998; Ma *et al.*, 2022), but they also require breeders to allocate substantial time and energy to carcass preparation, defense, and maintenance (Scott, 1998; Ma *et al.*, 2022; Potticary *et al.*, 2024). A breeding pair typically struggles to fully prepare a large carcass and often fails to completely consume it (self‐inspection; see Gray *et al.*, 2018). Subsequently, large carcasses are often poorly prepared (partial fur removal with limited oral and anal secretions covering it), making them more vulnerable to microbial colonization. This can negatively impact reproduction and offspring fitness by (a) reducing the nutritional value of carcasses, (b) producing harmful microbial metabolites, and (c) attracting competitors and scavengers through volatile emissions (Körner *et al.*, 2023). Hence, beetles breeding on large carcasses may allocate more energy toward self‐maintenance, such as feeding from the carcass, rather than investing more in offspring care and carcass maintenance. The comparable reproductive success between beetles breeding on large versus small carcasses (except for brood mass) supports this explanation.

### The presence of intraspecific competition has positive effects on male and female parental care

As predicted, both males and females increased their parental care in the presence of intraspecific competition, and such adjustments in care were consistent between sexes. These results indicate that in the presence of intraspecific competition, focal males and females likely spend more time in brood guarding and thereby may avoid the risk of infanticides by others (e.g. co‐breeders) (Bartlett, 1987a; Benowitz *et al.*, 2013; Komdeur *et al.*, 2013; Ma *et al.*, 2022), leading to an increased coordination in parental care between sexes (Müller *et al.*, 1990a; Trumbo, 2006; Ratz *et al.*, 2022). This finding aligns partially with previous research in *N*.* vespilloides*, where dominant males prolonged their stay with broods under intrasexual competition (Hopwood *et al.*, 2015). Similarly, in the bark beetle *Ips typographus*, high population density during breeding (i.e. intraspecific competition) affects parental care strategies (Anderbrant *et al.*, 1985). Unlike previous studies (Ratz *et al.*, 2022), in our study, an additional pair of beetles was introduced, and none of the beetles were allowed to escape from the breeding containers during breeding, more closely simulating natural conditions. This experimental design may have increased the opportunity for the smaller pair to access the carcass for food, and to produce their own offspring. Moreover, *N*.* vespilloides* is not a monogamous species, causing uncertain paternity and maternity in the developing larvae (Eggert & Müller, 1992; Müller *et al.*, 2007; Ma *et al.*, 2022). Hence, our results demonstrate that the focal males and females are likely to allocate more time to accessing and defending the carcass to safeguard their offspring. This behavior could also reduce the risk of infanticide by co‐breeders and enhance the likelihood of securing current reproductive success (Bartlett, 1987a; Müller & Eggert, 1990; Scott, 1998; Benowitz *et al.*, 2013; Ma *et al.*, 2022). We found no difference in reproductive success between the control and the intraspecific competition group, suggesting that increased parental care may offset the negative effects of conspecific intruders on reproductive success.

In nature, high competition from intraspecific individuals always indicates a high local population density and relatively reduced reproductive opportunities as the result of limited resources for each individual (Bartlett, 1987a; Jenkins *et al.*, 2000). This also demonstrates that males are likely to spend more time on carcasses for their own benefits, such as consuming the carcass and guarding their mates. Another possible explanation for the increasing care in both males and females is that good body condition (i.e. increased body weight) and/or having extended experience with offspring care after current breeding could help them outcompete others for future mating and breeding opportunities (e.g. Beeler *et al.*, 2002; Wang *et al.*, 2021). However, this hypothesis requires more research, as males and females would be expected to always increase their care whenever they have the opportunity to breed. We suggest further research to explore issues of reproductive allocation and mate choice in this species.

### The presence of interspecific competition has negative effects on male and female parental care

Contrary to our prediction, both males and females in the interspecific competition group decreased their parental care compared with those in the control group. Similar effects of interspecific interactions on parental care have also been observed in various other species (e.g. Requena *et al.*, 2009; Grayson *et al.*, 2013; Burdick & Siefferman, 2020; but see Sun *et al.*, 2014; Liu *et al.*, 2020; Tsai *et al.*, 2020). Blowflies are one of the most common competitors of burying beetles in nature (Scott, 1998; Sun *et al.*, 2014). They often locate carcasses faster than other species, oviposit rapidly, and their maggots both consume carcasses and produce secondary metabolites that degrade carcass quality (Vogel *et al.*, 2017; Shukla *et al.*, 2018; Chemnitz *et al.*, 2020). In response to the presence of interspecific competition with maggots, both males and females may reduce their investment in parental care for several reasons. First, the presence of maggots significantly reduces the nutritional values of carcasses, leading to both males and females abandoning broods (Pilakouta *et al.*, 2016). Second, the presence of blowfly maggots normally signals a high risk of reproductive failure (Sun *et al.*, 2014), which may prompt the beetles to conserve resources for future breeding attempts. Our findings partly align with previous work showing that *N*.* vespilloides* males desert their brood earlier when phoretic mites *Poecilochirus carabi* are present (De Gasperin *et al.*, 2015).

Consistent with this reduction in parental care, interspecific competition negatively affects reproductive success, with significantly smaller broods and lighter brood mass compared with the control groups. This outcome may be attributed to the observed reduction in parental care (Müller *et al.*, 1990b; Sahm *et al.*, 2023). Additionally, both males and females in the interspecific competition group gained more body mass after breeding than those in the control group, despite them being expected to spend more time and energy removing maggots and cleaning carcasses. However, this finding suggests that they may also prey the maggots. In this study, the introduction of blowfly maggots on carcasses caused dramatic reproductive failure for beetles (i.e. only 7 out of 33 broods had larvae that survived to dispersal) and low reproductive success. These findings suggest that under high competitive pressure and reduced resource quality, beetle parents minimize investment in current broods by providing less care, feeding opportunistically on carcasses and maggots, and prioritizing future reproductive success (Richardson & Smiseth, 2020; Wang *et al.*, 2022).

### Ecological and social factors lead to variation in parental care and parental coordination between sexes

We found that male and female care were positively correlated on large carcasses but not on small ones, suggesting that carcass size influences parental coordination strategies in care. On large carcasses, pairs appear to adopt a matching strategy, whereas on small carcasses they seem to use a sealed‐bid strategy (Houston & Davies, 1985; Johnstone & Hinde, 2006; Boncoraglio & Kilner, 2012). Such an adjustment in strategies depending on resource size is suggested to resolve sexual conflicts over parental care. Similar patterns of parental coordination within breeding pairs are also observed in some avian species, such as Lesser Black‐backed Gull *Larus fuscus* (Kavelaars *et al.*, 2021) and Manx Shearwater *Puffinus puffinus* (Gillies *et al.*, 2022). Additionally, we found a positive correlation between changes in parental body mass and the levels of parental care, suggesting that access to the carcass benefits parents by allowing them to feed on it, potentially saving more resources for future reproduction (Wang *et al.*, 2021). However, this case is more common in males than in females, highlighting sex differences in the costs and benefits of parental care. For example, males are more likely to invest in maintaining their condition for future mating opportunities (Ma *et al.*, 2022). When resources are abundant, a positive correlation in male and female care may arise due to reduced sexual conflict, despite the fact that biparental care does not enhance offspring fitness. Under such conditions, biparental care may be evolutionarily stable because the unrestricted access to the carcass of females and males minimizes competition over resources and reproduction between them. In contrast, when resources are limited, males and females seem to invest independently, where females work near their physical limits to maximize reproductive success (Wang *et al.*, 2021), while males may adjust their level of care more flexibly (Smiseth *et al.*, 2005). Our study demonstrates the strength of the correlation in the level of care between sexes varying across contexts, but it can only to a limited extent explain how parental beetles adopt different strategies in response to their breeding partners. More studies employing manipulation experiments, such as handicap studies, are needed to address this question.

Similar to the control group, we observed a positive correlation between male and female parental care in the presence of intraspecific competition, suggesting that parents maintained a matching strategy to mitigate sexual conflicts over parental care in such a situation (Johnstone & Hinde, 2006). This finding is consistent with results in the dung beetle *Onthophagus taurus*, which reported a positive correlation between male and female caregiving investments (Hunt & Simmons, 2002). However, our findings contradict those of a study on *N*.* vespilloides*, which observed a negative relationship between male and female provisioning (using a mate removal design, Smiseth & Moore, 2004). We propose that the presence of conspecific competitors during the breeding phase enhances cooperation between male and female care, promoting the evolution of biparental care (Trumbo, 2012). This coordination of care may help parents to address challenges in resource defense and provisioning, ultimately improving reproductive success (Richardson & Smiseth, 2019; Ma *et al.*, 2022). Interestingly, the similarity in parental coordination between the control and intraspecific competition groups suggests that intraspecific competition does not influence the resolution strategies for sexual conflict over parental care.

In contrast, in the interspecific competition group, no significant correlation was found between male and female care, suggesting a sealed‐bid strategy (Houston & Davies, 1985). The presence of blowfly maggots likely intensified resource limitations because maggots consume a significant amount of the carcass, promoting both parents to focus individually on carcass maintenance tasks such as maggots removal. Although paired beetles are generally more effective at maggots removal than solitary individuals, there may not be coordinated investment in maggots removal between sexes (but see Tsai *et al.*, 2020). The absence of parental coordination under interspecific competition suggests that resource constraints and reproductive failure risk decrease cooperation (Scott, 1998; Johnstone & Hinde, 2006; Alonso‐Alvarez & Velando, 2012; Trumbo, 2012). The presence of blowfly maggots leads to high reproductive failure, making each parent prioritize self‐maintenance over cooperative brood care or defense. Specifically, males may benefit from feeding themselves on the carcass while females benefit from assessing the risk of reproductive failure to abandon the brood at the appropriate time. One limitation of this study is that parental care measurements combined direct and indirect care, measured by the frequency of parental individuals being present on or in the prepared carcasses. More detailed behavioral observations are needed to further explore parental care strategies during breeding in this species.

## Conclusion

In conclusion, our results in *N*.* vespilloides* demonstrate that resource availability and both intraspecific and interspecific competition resulted in varied reproductive costs and benefits, prompting sex‐specific parental strategies and coordination. Compared with the control group (large carcass, no competition), males maintained the levels of care on small carcasses, whereas females increased care. Both sexes elevated care under intraspecific competition but reduced care under interspecific competition. Moreover, ecological and social factors influenced how parental beetles resolved sexual conflicts over parental care. On large carcasses and under conditions of intraspecific competition, parents adopted the matching strategy to coordinate care. Conversely, on small carcasses and in the presence of interspecific competition, parents employed the sealed‐bid strategy. Overall, these findings demonstrate that resource availability and competitive pressures are critical drivers shaping the evolution of parental care coordination strategies, serving as key selective forces promoting biparental care.

## Author contributions

L.M. and J.K. contributed to the study conception and design. L.M. performed the experiments. D.M. analyzed the data and wrote the first draft of the manuscript. L.M. and J.K. led the editing of the manuscript. All authors read and approved the final manuscript.

## Disclosure

There is no conflict of interest to declare for this study.
